# Social Media Approval Reduces Emotional Arousal for People High in Narcissism: Electrophysiological Evidence

**DOI:** 10.3389/fnhum.2019.00292

**Published:** 2019-09-20

**Authors:** Kyle Nash, Andre Johansson, Kumar Yogeeswaran

**Affiliations:** ^1^Department of Psychology, University of Alberta, Edmonton, AB, Canada; ^2^Department of Psychology, University of Canterbury, Christchurch, New Zealand

**Keywords:** narcissism, arousal, social media, selfie, event-related potential

## Abstract

We used event-related potentials (ERPs) to examine if posting a “selfie” and receiving validation from others in the form of “likes” on social media can help narcissists reduce psychological distress. After all participants completed the narcissistic personality inventory (NPI) and experienced social exclusion, participants completed an auditory startle task that elicits the P3 to white noise—an ERP component that reflects emotional arousal and is sensitive to psychological distress. Participants were then randomly assigned to either view a personal “selfie” that quickly received a significant number of ostensibly real “likes” (*selfie with likes* condition), view a “selfie” with no feedback (*selfie only* condition), or view a neutral picture before (*neutral picture* condition) completing the auditory startle task again. Results revealed that participants high on the Leadership/Authority subscale of the NPI in the “selfie” with “likes” condition demonstrated a pre–post manipulation decrease in P3 mean amplitude, relative to participants in the other two conditions. These results suggest that approval *via* social media can help certain kinds of narcissists alleviate distress from social exclusion.

## Introduction

Social connection is a fundamental psychological need (Leary and Baumeister, 2017). People who feel connected are healthier, happier, and live longer (for a review, see Hawkley and Cacioppo, 2010). Social exclusion, on the other hand, can cause emotional pain, reduce emotional sensitivity, increase aggression, and lead to long-term psychological problems (for reviews, see Baumeister et al., 2007; Williams, 2007). For narcissists, social connection is a particularly thorny problem. Narcissists prioritize social validation and superiority in social interactions, but have difficulty forming genuine, close, and stable relationships (Morf and Rhodewalt, 2001; Bergman et al., 2011). Initially seen as charming, the narcissist’s myopic pursuit of approbation leads others to find the relationship taxing and one-sided (Campbell and Campbell, 2009). Narcissists can seek short-term social connections for validation, such as one-night stands and casual sexual relationships (Jonason and Buss, 2012). However, short-term connections are costly and not reliably rewarding. Thus, narcissists face a dilemma: social connections are their primary source of validation, but pursuing these asymmetric relationships may not always be feasible.

However, the ways in which we establish and maintain relationships has been greatly impacted by social media. Online platforms afford instant connection with family, friends, and acquaintances. Social media can be psychologically rewarding. For example, social media use among people with large social networks causes a temporary bump in self-esteem (Wilcox and Stephen, 2012). Additionally, the number of “likes” a photograph uploaded to Instagram receives is associated with increased activation in the nucleus accumbens, a brain region integral to reward-related processing (Sherman et al., 2018).

Could social media have handed narcissists the perfect solution to their dilemma? That is, might validation through social media allow narcissists to meet their deep need for social approval without the effort of real-world relationships? A central psychological currency on social media is validation. Social networking sites such as Facebook and Instagram allow users to enhance their photos using filters and editing tools before uploading to a potentially global audience who can provide instant, positive feedback. And the obstacles in establishing and maintaining genuine connections are avoided. Research shows that narcissists are particularly active on social media (Buffardi and Campbell, 2008). Narcissists also report more frequent “selfie” (i.e., picture of oneself) posting (Barry et al., 2017), higher levels of self-reported attractiveness in “selfies” (Re et al., 2016), and increased positive affect when posting selfies, particularly among those with high levels of grandiose narcissism (McCain et al., 2016). Social media may thus be an ideal hunting ground for narcissists to pursue reliable shots of validation. To date, however, no research has demonstrated that social media serves a psychological function for narcissists. The present research examines if people high in narcissism can regulate negative affect from exclusion through validation from social media.

## Narcissism, Exclusion, and the Brain

We focus on narcissism as conceptualized in social-personality (SP) psychological research—a personality trait that varies normally across individuals—as opposed to conceptualizations of narcissism as a personality disorder (Cain et al., 2008). Trait narcissism is characterized by a grandiose yet fragile self-image sustained by external validation and approval. Threats to this self-image cause narcissists to react with increased anger and aggression (Bushman and Baumeister, 1998). As part of intrapsychic and interpersonal strategies employed to attain social approval or superiority, narcissists seek physical attractiveness, personal success, and social dominance (Campbell and Campbell, 2009). After receiving social approbation, however, narcissists become particularly callous, displaying particularly low levels of empathy (McGregor et al., 2013). At a trait level, narcissism is associated with high levels of extraversion and agency and low levels of agreeableness. Men tend to be more narcissistic (though this may be due to shared variance with psychopathy), trait levels decline with age, and culture moderates perceptions of narcissism (Wilson and Sibley, 2011; Grijalva et al., 2015). Interpersonally, narcissism is characterized by poor regard for others and poor relational functioning (Konrath and Bonadonna, 2014). Narcissists feel entitled to special treatment and they owe others little (Millon and Davis, 1996).

Narcissists are particularly sensitive to exclusion. For example, social exclusion causes narcissists to react more aggressively toward those who rejected them and even toward uninvolved third parties (Twenge and Campbell, 2003). On the other hand, narcissists reliably report higher levels of self-esteem, happiness, and well-being, leading some researchers to reasonably conclude that narcissism is psychologically healthy (Sedikides et al., 2004). Such conclusions contrast sharply with classical views on narcissism as a compensatory defense against a deeper sense of insecurity or inferiority (Freud, 1914; Adler, 1939; Horney, 1945/2013; Bowlby, 1988/2012). Would one expect a narcissist to self-report anything other than cheery prospects and sanguine psychological states?

Contemporary research using more objective neuroscience measures support these classical views (see also Millon and Davis, 1996, for a biosocial approach to narcissism). For example, in a recent fMRI study, participants viewed pictures of their own face, a friend’s face, and a stranger’s face. In contrast to the self-report research on selfies and narcissism cited above (McCain et al., 2016), results here showed that viewing their own face caused narcissistic men to demonstrate increased activation in the anterior cingulate cortex (ACC) and the right anterior insula, a pattern of activation consistent with negative affect or emotional conflict (Jauk et al., 2017). This finding stands in direct contrast to the idea that narcissists find the self inherently rewarding and points to an implicit sense of insecurity. Similarly, a diffusion tensor imaging study found that narcissism is associated with reduced connectivity between the ventral striatum and the medial prefrontal cortex (mPFC; Chester et al., 2015). The mPFC has been associated with self-relevant processing and the ventral striatum is associated with reward-related processing. Further, connectivity between these two regions has been associated with increased positive self-regard (Chavez and Heatherton, 2014). This suggests that narcissism also involves a restricted neuroanatomical link between the self and reward.

In response to social exclusion, narcissism was associated with increased activation in the putative “social pain” network, which includes the ACC and the anterior insula (Cascio et al., 2014). Interestingly, self-report indices did not reveal any association between narcissism and distress after social exclusion (Cascio et al., 2014). Moreover, aggressive reactions to social exclusion characteristic of narcissists are moderated by the degree of activation in the ACC (Chester and DeWall, 2016). The authors reasoned that the narcissist’s distress was caused by detection of a discrepancy between the idealized self and the threatened self. Increased distress then led to increased aggression among narcissists. In all, these findings support the notion that narcissists are particularly vulnerable and highly motivated to defend against social exclusion. More broadly, these findings demonstrate how neuroscience measures can directly address the inherent problems in using self-report to reveal underlying mechanisms in narcissism-related processes.

## The Current Study

To avoid the issues inherent in measuring the relationship between narcissism and distress to social exclusion using self-report, the current study used objective, electrophysiological measures. To create distress, all participants first experienced social exclusion *via* the well-established Cyberball task (Williams and Jarvis, 2006; Hartgerink et al., 2015). Participants were then randomly assigned to one of three conditions: in the “selfie with likes” condition; participants took a “selfie,” edited and enhanced the photo to their liking, uploaded the “selfie” to their Instagram account, and then viewed bogus, but ostensibly real feedback in the form of “likes” on their Instagram account. The bogus feedback was delivered by a novel software program developed for this study. In the “selfie” only condition, participants similarly uploaded an edited “selfie” to their Instagram account, but received no feedback from others and only viewed the picture for the same duration. This condition was included to explore whether social approval was necessary to mask distress following social exclusion or whether self-presentation alone was sufficient. Finally, the control condition involved viewing a motivationally/affectively neutral image (i.e., gravel, see Harmon-Jones and Gable, 2009) on Instagram and involved no “selfie” or validation from others.

Both before and after uploading the selfie onto Instagram, participants completed a passive-listening auditory startle task, during which EEG was recorded. Infrequent, aversive blasts of white noise (i.e., startle stimuli) elicit a characteristic event-related potential (ERP) component—variously termed the P3a, startle P3, or novelty P3 (Combs and Polich, 2006; Keil et al., 2007). The P3 or P3a component elicited by white noise or acoustic startle peaks over fronto-central electrodes between ∼250 and 350 ms post-stimulus and is thought to reflect rapid, automatic shifts in attentional processes toward motivationally salient stimuli (Polich, 2007; Volpe et al., 2007; Frank et al., 2012). According to the locus coeruleus-norepiniphrine (LC-NE) hypothesis of the P3 (Nieuwenhuis et al., 2005), the P3 is driven by the LC-NE system and represents the cortical analog of emotional arousal. The LC-NE system is integral to emotional arousal (Gray and McNaughton, 2000; Berridge and Waterhouse, 2003; Ulrich-Lai and Herman, 2009). The LC is activated by the same stimuli and is modulated by the same precedent conditions as the P3 (e.g., in an oddball paradigm, Nieuwenhuis et al., 2005, 2011) and direct stimulation of the LC-NE system causes a P3-like component (Vazey et al., 2018). Further, P3 amplitude tracks measures of sympathetic arousal. For example, skin conductance correlates with novelty and P3a components in auditory oddball tasks (Rushby et al., 2005; Rushby and Barry, 2009). Further, the sympathetic arousal component in pupil dilation to novel sounds is associated with P3 amplitude to the same stimuli (Widmann et al., 2018).

Importantly, the P3 is sensitive to distress or negative affect. For example, stressful or anxiety-provoking events cause increased P3 amplitude on a subsequent auditory oddball task (Grillon and Ameli, 1994; Ermutlu et al., 2005). High-anxiety people show the largest P3a, particularly after negative affect induction (Wang et al., 2017a). Increased P3 amplitude to white noise is associated with increased self-reported negative affect (Masuda et al., 2018). Enhanced P3a amplitudes are associated with disorders involving increased negative affect, including panic disorder (Clark et al., 1996), obsessive–compulsive disorder (Ischebeck et al., 2011), and post-traumatic stress disorder (PTSD) (Kimble et al., 2000). Finally, a drug that increases NE release causes increased P3a amplitude (Missonnier et al., 1999). Conversely, meditation reduces the amplitude of the P3 to white noise burst (Cahn and Polich, 2009). A low dose of alcohol mutes the P3 to novelty (Marinkovic et al., 2001). Clonidine, a drug that attenuates baseline NE activity, decreases the amplitude of the P3a to auditory oddball stimuli (Brown et al., 2015). Sensation seeking, a trait characterized by low negative affect and low distress, correlates with decreased P3 (Wang and Wang, 2001).

In sum, the P3 directly reflects emotional arousal and is sensitive to distress, suggesting that P3 mean amplitude to white noise bursts may be used as a more objective and direct measure of emotional arousal to social exclusion. Consistent with this, social exclusion causes increased P3a amplitude, and P3a amplitude is related to the negative mood associated with social exclusion (Gutz et al., 2011). A more objective measure is a requirement in studies on narcissism and emotional reactions to ostracism. As noted above, a narcissist’s social pain to rejection can only be seen “in the brain” (Cascio et al., 2014), and this pain drives compensatory behavior for people high in narcissism, while self-report is unrelated to the degree of social pain or compensatory behavior. Our pre–post measure of white noise P3 amplitude thus allowed us to sidestep issues with self-report and directly examine if narcissists, driven by a sharpened need to obtain social validation after exclusion, would show the greatest reduction in emotional arousal in the “selfie with likes” condition.

## Method

This study received ethical approval from the University of Canterbury Human Ethics Committee. Based on a meta-analysis that determined that the effect of Cyberball exclusion on intrapersonal variables (i.e., mood and arousal) was large (overall effect size, Cohen’s *d* > 1; see Hartgerink et al., 2015), we aimed to include 30 individuals per condition (according to analyses calculated in G^∗^Power statistical package: many groups ANOVA main effects and interactions *F*-test, *expected* effect size *f* = 0.5, *alpha* = 0.05, *power* = 0.95, and *number of groups* = 3, *output sample size* = 54 for 95% power, achieving greater than 95% power for detecting differences in distress. Alternatively, for an interaction effect: linear multiple regression *R*^2^ increase *F-*test, *expected* effect size *f*^2^ = 0.15, *alpha* = 0.05, *power* = 0.95, and *number of tested predictors* = 1, *total number of predictors* = 3, *output sample size* = 89 for 95% power in detecting interaction effects). We ran until the end of the semester. Eighty-three undergraduates participated in the study for course credit. Six participants were excluded for having too many artifacts (<12 artifact-free startle trials), leaving 77 participants for data analysis (18 male, 59 female; *M*_age_ = 20.8, *SD* = 3.73 years).

### Procedure

Participants were required to bring their cell phones with the Instagram app installed on it to take part in the study. On arriving, all participants first proved written informed consent. They were fitted with a 14-electrode, quick application EEG headset (Emotiv EPOC+, Emotiv Systems, Inc., San Francisco, CA, United States). All materials were completed on a computer. Participants first answered demographic questions and several personality questionnaires (all data available upon request), including the 40-item narcissistic personality inventory (NPI, Raskin and Terry, 1988). All participants then experienced social exclusion using the Cyberball paradigm. After completing a pre-measure passive auditory startle task, participants were randomly assigned to one of three picture viewing conditions: a *selfie with likes* condition, a *selfie only* condition, and *neutral picture* condition (control condition). Participants then completed a post-measure startle task and items probing suspicion and conscientiousness. Finally, participants were fully debriefed and thanked for their time.

### Cyberball Exclusion

Participants were led to believe that they were playing a game of virtual catch over the Internet with two other ostensibly “real” players. Participants entered a user name that was supposedly visible to the other players. All participants experienced Cyberball exclusion. Participants were initially included in the virtual game of catch (first 6 passes) but were then excluded from passes from the other two players for the remainder of the game (the remaining 19 passes), thus receiving the ball only 8% of the time. Past research shows that Cyberball exclusion causes heightened negative affect and defensive behavior (Gerber and Wheeler, 2009). Narcissists are particularly vulnerable to this experience, demonstrating increased activation in the putative social pain network (Cascio et al., 2014) and increased defensiveness (Chester and DeWall, 2016). Based on this reliable effect among people high in narcissism, we did not include a social inclusion manipulation for comparison in order to have a more manageable research design.

### Selfie Manipulation

After the first startle task, participants were randomly assigned to one of three conditions: the *selfie with likes* condition, the *selfie only* condition, or the *neutral picture* condition.

In the *selfie with likes* condition, participants were instructed to take and upload a “selfie” to their Instagram account, and include the following hashtags: #selfie #fitspo #fitspiration. They were informed that the purpose of this exercise was to examine how the use of highly popular hashtags, guaranteed to get high exposure on Instagram, influenced social feedback in the form of “likes.” Participants were taken to a side room and were given time to take and edit a “selfie” on their phones. They showed the experimenter prior to uploading the image to make sure the hashtags and sizing were correct. Participants then uploaded their “selfie” and handed their phone to the examiner to avoid distraction throughout the remainder of the study. Participants then returned to their computer and opened the “selfie” on Instagram online. They read an information sheet explaining that a screen refreshing software would start and they could observe how many likes they received. In reality, a transparent screen developed for the study was opened over top of the current Instagram display. This screen then generated fake likes on the participant’s Instagram photos. The screen would appear to refresh every 10 s displaying a spinning loading wheel and the Instagram “likes” would gradually increase. All participants saw the same pattern and number of “likes” (26) over a span of 6 min. The participant signaled the experimenter when automatically prompted by the computer after 6 min.

In the *selfie only* condition, participants uploaded a new “selfie” to their Instagram account following the same procedure. However, upon uploading the “selfie,” they were told that would merely be viewing the picture for approximately 5 min. No “likes” were provided during this time. The participant also signaled the experimenter when automatically prompted after 6 min. In the *neutral picture* condition, participants did not take or upload a “selfie” to Instagram. Instead, these participants observed a picture of gravel on a dummy Instagram account for 6 min before moving on to the next task. A picture of gravel was used to ensure that participants did not find the experience rewarding or reflect upon the self. These conditions thus allowed us to determine if any effect on P3a mean amplitude are due to the “likes” themselves, or merely viewing a picture of the self.

### Measures

#### Narcissism (NPI)

As part of a larger package of questionnaires (to help ensure people did not connect narcissism items with selfie posting), participants scored each item from the NPI on a scale of 1 (strongly disagree) to 5 (strongly agree). In analyzing this scale, we followed the recommendations made by Ackerman et al. (2011) on NPI use. These authors demonstrated that an NPI total score conflates meaningfully different aspects of personality and a three-factor solution provides more accurate demonstrations of psychological processes in narcissism. We computed and examined the same three facets of narcissism (see Ackerman et al., 2011, for full description of facets), including “Leadership/Authority” (L/A, e.g., “If I ruled the world it would be a better place”; α = 0.835), “Grandiose Exhibitionism” (GE, e.g., “I like to look at my body,” α = 0.739), and “Entitlement/Exploitativeness” (E/E, e.g., “I will never be satisfied until I get all that I deserve,” α = 0.566). We used the NPI given its widespread use in SP research (Cain et al., 2008). Notably, this three-factor solution has been demonstrated as reliable and valid (Ackerman et al., 2018), including in research on selfie posting and narcissism (Weiser, 2015). As in past research, men in our sample demonstrated higher levels of narcissism on each subscale (all *p’*s < 0.05).

### Auditory Startle Paradigm

Following Cyberball, participants put on headphones (volume setting 50 in Windows) and listened passively to a series of beeps (pure 1000 Hz tones for 50 ms) and white noise blasts. The ratio of startling noises to beeps was 2:8. Each stimulus was presented for a second and the entire paradigm lasted for 3 min, for a total of 180 trials (approximately 36 static and 144 beep trials). To minimize movement, participants were asked to fixate on a small cross presented on the computer screen during the task. This task was presented both before and after the manipulation. We focused on the P3 ERP component to white noise as an index of emotional arousal after a distressing event—social exclusion. Recall that the P3 is enhanced during social exclusion and the P3 is related to negative mood following Cyberball exclusion (see Wang et al., 2017b). Further, the link between P3 and emotional arousal is strongest at frontal nodes (Ermutlu et al., 2005), in passive auditory oddball tasks (Grillon and Ameli, 1994; Rushby and Barry, 2009), in response to highly deviant and expectancy violating stimuli (Nieuwenhuis et al., 2011; Frank et al., 2012), and in relation to the P3a or novelty P3 component (Missonnier et al., 1999; Brown et al., 2015; Wang et al., 2017a, b; Widmann et al., 2018). Consequently, to provide the best index of emotional arousal, we focused on the P3 to infrequent white noise bursts in a passive auditory oddball task, measured at frontal nodes.

### EEG Recording and Analyses

During both pre- and post-manipulation auditory startle tasks, electroencephalography (EEG) was recorded using a 14-channel (gold-plated contact-grade hardened copper with felt pads moistened with saline) Emotiv EEG wireless headset (Emotiv Systems, Inc., San Francisco, CA, United States) and Emotiv TestBench software at a sampling rate of 128 Hz. The 14 channels, AF3, AF4, F3, F4, F7, F8, FC5, FC6, P7, T7, T8, P8, O1, and O2, were positioned according to the 10–20 International System. Two mastoid electrodes were used as online reference. Validation research demonstrates that this headset system proves a reliable alternative to standard systems in measuring ERPs to auditory oddball stimuli, including measures of the N2 and P3 components at frontal electrodes (Badcock et al., 2013; Mayaud et al., 2013). Emotiv EEG technology has become an increasingly popular alternative to standard EEG systems in social and cognitive neuroscience research (Louwerse and Hutchinson, 2012; Steinhubl et al., 2015; Agroskin et al., 2016) and in brain–computer interface (BCI) applications (Bobrov et al., 2011; Debener et al., 2012; Choi and Jo, 2013; Khushaba et al., 2013; O’Regan and Marnane, 2013; De Vos et al., 2014; Vourvopoulos and Liarokapis, 2014).

**FIGURE 1:**
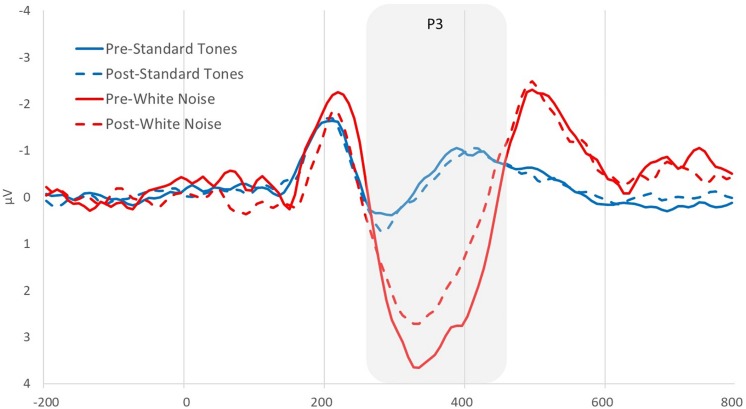
Pre- and post-picture viewing manipulation grand average event-related potentials (ERPs) at electrode F4 for standard tones and white noise stimuli.

Using the analysis software Brain Vision Analyzer 2.0 (BVA 2), EEG data from both recordings was band-pass filtered between 0.1 and 30 Hz. Blinks were statistically removed using the automatic ocular independent component analysis (HEOG and VEOG reference electrode = AF3) in BVA 2, which isolates and deletes blink-related factors. Artifacts were then automatically detected and removed using the following parameters −100 to +100 μV min/max threshold, 50 μV maximum voltage step, 0.5 μV lowest allowed voltage (maximum–minimum) in 100-ms intervals. Data were segmented into 1000-ms epochs locked on either beep or white noise presentation, 200 ms before to 800 ms after the stimulus. All artifact-free epochs were then averaged, creating average ERPs of beeps and startle tones for each participant. Each average ERP was baseline-corrected by subtracting the average voltage during the 200–0 ms time period prior to the stimulus. The white noise P3 was quantified for both beeps and startle stimuli as the mean positive amplitude between 275 and 450 ms after stimulus at site where the startle component was maximal, the fronto-central electrode F4 (see Figure 1). A P3 change score was computed [deltaP3 or (D)P3 = Auditory Oddball Time 2 - Time 1] to determine the degree to which people showed a decrease in P3 amplitude as a function of condition and narcissism levels. A P3 difference score (P3diff = white noise - standard tone) was also computed to remove processes common to both ERPs and isolate an error-specific variable (Luck, 2014). Finally, a P3diff change score [(D)P3diff = Auditory Oddball Time 2 - Time 1] was computed to ensure that changes in P3 were specific to white noise stimuli. Note that all variables proved normal in terms of skewness and kurtosis (George and Mallery, 2010). Further, all necessary assumptions for moderation analyses were met, including those related to multicollinearity, independence of residuals, homoscedasticity, normality of residuals, and evidence that no influential cases biased the data.

## Results

**FIGURE 2:**
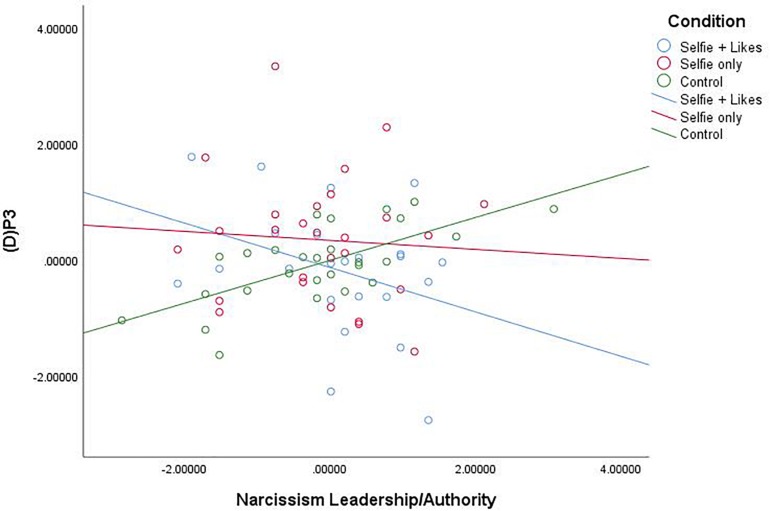
Scatterplot for the interaction between condition and Narcissism—Leadership/Authority (*Z* score) on white noise P3 mean amplitude change scores (*Z* score).

Our primary research question is based on the idea that social media can fulfill a narcissistic need, and we expected that people who were high in narcissism would be the most prone to these effects. We had no expectations for main effects or effects at other levels of narcissism. Thus, we conducted moderated multiple regression analyses using Hayes (2013) PROCESS macro (Model 1, 5,000 bias-corrected bootstrapped resamples) with X = condition (coded as X1: 0 = selfie with likes, 1 = selfie only, 0 = neutral picture; and X2: 0 = selfie with likes, 0 = selfie only, 1 = neutral picture), W = narcissism subscale, and Y = (D)P3 for each narcissism subscale, Bonferroni corrected for multiple tests (α = 0.05/3 = 0.0167). Results revealed that the narcissism facet L/A interacted with condition to impact (D)P3, *F*(2,75) = 5.027, *p* = 0.009, *R*^2^ = 0.117 (see Figure 2). Comparison of estimated conditional means (ECM, at the 84th percentile for high and 16th percentile for low levels of narcissism) revealed that at high levels of the L/A facet of narcissism (84th percentile = 3.50), people in the *selfie with likes* condition (ECM = −2.486) showed a significant decrease in (D)P3 mean amplitude compared to those in the *selfie only* condition (ECM = 0.905), *t*(76) = 2.062, *p* = 0.043, CI [0.111, 6.671] and the *neutral picture* condition (ECM = 1.303), *t*(76) = 2.411, *p* = 0.019, CI [0.656, 6.923]. At low levels of the L/A facet of narcissism (16th percentile = 2.20), people in the *selfie with likes* condition (ECM = 1.745) showed a significant *increase* in (D)P3 mean amplitude compared to only those in the *neutral picture* condition (ECM = -2.784), *t*(76) = 2.223, *p* = 0.0029, CI [−8.589, −0.467], but there was no difference between the two selfie conditions at low L/A (*p* = 0.994).

**FIGURE 3:**
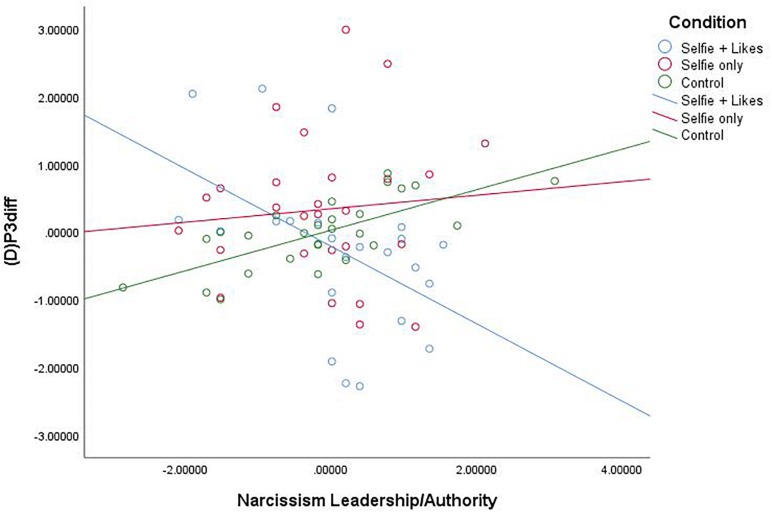
Scatterplot for the interaction between condition and Narcissism—Leadership/Authority (*Z* score) on white noise P3diff mean amplitude change scores (*Z* score).

Similar analyses also revealed that the narcissism facet L/A interacted with condition to impact (D)P3diff, *F*(2,71) = 7.005, *p* = 0.002, *R*^2^ = 0.154 (see Figure 3). As above, comparison of estimated conditional means revealed that at high levels of L/A, people in the *selfie with likes* condition (ECM = -4.535) showed a significant decrease in (D)P3 mean amplitude compared to those in the *selfie only* condition (ECM = 1.991), *t*(76) = 3.376, *p* = 0.001, CI [2.466, 9.586] and the *neutral picture* condition (ECM = 0.871), *t*(76) = 3.170, *p* = 0.002, CI [2.006, 8.808]. This demonstrates that these differences are due to processes specific to the startle trials. Again, at low levels of the L/A facet of narcissism, people in the *selfie with likes* condition (ECM = 2.575) showed a significant *increase* in (D)P3 mean amplitude compared to only those in the *neutral picture* condition (ECM = −2.850), *t*(76) = 2.455, *p* = 0.017, CI [−9.832, −1.018]. There was again no difference between the two selfie conditions at low L/A (*p* = 0.320). Further, though men demonstrated higher levels of narcissism on each subscale, including gender as a covariate in each of the above analyses did not change the results. Notably, *post hoc* power analysis, calculated in G^∗^Power, revealed that observed power = 0.917.

Finally, the same analyses for both the GE and E/E facets demonstrated non-significant interactions for both the (D)P3 and (D)P3diff scores (all *p*s > 0.520). Thus, the current results are specific to the L/A facet of narcissism, as measured with the NPI.^1^

## Discussion

The current study used a multidisciplinary, multimethod approach to examine if people high in narcissism regulate distress through social media approval. We designed a novel software to provide ostensibly genuine “likes” on real “selfie” photos posted on valid Instagram accounts. All participants first experienced the negative emotional event of social exclusion *via* the Cyberball task (Williams and Jarvis, 2006). After exclusion, participants were randomly assigned to either view a real, personal “selfie” that quickly received a significant number of likes, view a “selfie” with no feedback, or view a neutral picture. To measure distress (and avoid the self-presentation and bias concerns that go hand in hand with narcissism research), we indexed the P3 mean amplitude to aversive, unexpected bursts of white noise. This ERP component is related to emotional arousal and is heightened by distressing events (Masson and Bidet-Caulet, 2018), including distress after social exclusion (Gutz et al., 2011). The auditory startle task was administered both before and after the manipulation to index P3 mean amplitude changes.

We found that participants high in the narcissism subscale L/A that received “likes” on a “selfie” showed the largest pre–post decrease in P3 mean amplitude and the P3 difference wave mean amplitude, compared to people high in L/A in the other conditions. The other narcissism subscales, GE and E/E, did not interact with condition to predict change in P3 amplitude. These results suggest that, for people with a stronger sense of leadership ability and dominance, social media validation reduced distress caused by social exclusion. The L/A facet is viewed as the more adaptive aspect of the NPI (Ackerman et al., 2011). This suggests that current results may reflect a more adaptive regulatory process. This could also partially explain why narcissists are more active on social media (Buffardi and Campbell, 2008) and report more frequent “selfie” posting (Barry et al., 2017). Furthermore, these results support the idea that social media may provide certain (but not all) narcissists with an ideal solution to their unique social dilemma. It allows them to reliably attain social validation without resorting to real-world relationships that typically require reciprocity and sacrifice.

The current study has certain limitations that may be addressed in future research. First, future work could add a comparison condition to the design and manipulate exclusion vs. inclusion to examine if heightened distress among people high in narcissism in the exclusion condition only shows reduced distress to approval on selfie posting. Moreover, while the current research focuses on a social threat as narcissists are particularly sensitive to social exclusion, future work should also examine whether non-social threats (e.g., trying to solve challenging math problems) produce similar distress levels for people high in narcissism that can subsequently be alleviated by social media. Second, we did not include a self-report measure of distress because we felt self-report was uniquely ill-suited to the current research questions. This was based on research demonstrating that narcissism does not predict self-reported distress to social exclusion despite increased activation in the social pain network (Cascio et al., 2014) and increased defensiveness (Chester and DeWall, 2016). Further, including such a measure could inform the participant about our research questions and produce demand characteristics, particularly for the already defensive and biased individuals high in narcissism. However, at the end of the study, we did include a measure of meaning in life (Steger et al., 2006), which includes the meaning presence subscale. Meaning presence has been found to be correlated with low levels of anxiety and distress, and a lack of meaning has been found to be correlated with high levels of anxiety (Steger et al., 2008). Having this measure allowed us to examine the following: if white noise P3 amplitude is positively related to levels of distress, and meaning presence is negatively related to distress, then white noise P3 amplitude should be negatively related to meaning presence. Correlational analyses support this idea. The meaning presence subscale was negatively correlated with both Time 1 and Time 2 white noise P3 amplitudes (Time 1: *r* = −0.252, *p* = 0.027; Time 2: *r* = −0.261, *p* = 0.022). Considering these results and the facts that social exclusion reliably elicits negative affect (Hartgerink et al., 2015), the white noise stimulus is itself aversive (Masuda et al., 2018), and the LC-NE system is critically involved in distressed responses, the most parsimonious interpretation is that the P3 was sensitive to distress in this study. However, future work may address the current limitations associated with these kinds of inferences and use corroborative measures of emotional arousal. Finally, future research could benefit from adapting the current research questions to a within-subjects design.

Overall, however, our findings support the idea that social media may be a happy hunting ground for narcissistic needs. We found that validation through “likes” on a real “selfie” posted online reduced neural indices of emotional arousal after social exclusion for people high on the L/A subscale of narcissism. Such narcissists, who prioritize social superiority over relational harmony, face a dilemma. Pursuing asymmetric relationships for narcissistic needs can tend to disrupt those relationships. Social validation earned online might allow certain people high in narcissism to sidestep that dilemma and efficiently regulate distress.

## Data Availability

The datasets generated for this study are available on request to the corresponding author.

## Ethics Statement

This study received ethical approval from the University of Canterbury Human Ethics Committee. Written consent was obtained prior to participation.

## Author Contributions

All authors listed have made a substantial, direct and intellectual contribution to the work, and approved it for publication.

## Conflict of Interest Statement

The authors declare that the research was conducted in the absence of any commercial or financial relationships that could be construed as a potential conflict of interest.

## Footnotes

1 Conducting the same analyses using an NPI total score demonstrates comparable, but mostly non-significant, results.
